# Intercranial Tumor

**Published:** 1860-07

**Authors:** W. J. Moody


### ARTICLE 2.

### INTERCRANIAL TUMOR.

BY W. J. MOODY, M. D.

Edwin Williams, aged thirteen, form light and active, constitution robust, and health heretofore excellent, was afflicted about the first of May last, with a swelling on the left side of the neck, reaching from a point just posterior to the angle of the inferior maxilla, to one opposite to its articulation with the temporal bone. At first not much attention was paid to the tumor, but it soon became so intensely painful as to alarm the parents, and excite my unqualified surprise. I was thus induced to make a more thorough examination, when I ascertained that the tenderness extended to the articulation. I now became satisfied that the inferior maxillary nerve was involved. If I had any doubts of this, they were soon removed by the appearance of a tumor upon the inside of the mouth, exactly at the point of division of the nerve, by the external pterygoid muscle. This protuberance became extremely painful, and it was with the utmost difficulty that the patient could separate the jaws far enough to admit necessary food.

Emollient and anodyne poultices were now kept constantly applied, externally, with the effect of mitigating, somewhat, the pain and swelling, until, finally, about a fortnight from the time of the first attack of the disease, it was dislodged from its first point of attack, only to re-appear immediately at another.

The patient soon began to complain of pain above and posterior to the angle of the right eye. Soon the flesh began to puff up at the point of emergence of the frontal nerve through the supraorbital foramen, and extending throughout the whole course of the nerve. This attack was attended with the same severe pain as the original one, for a few days, when it, in its turn, disappeared, to be followed immediately by the grave symptoms of intercranial tumor. I shall, in the course of these remarks, enter into no disquisition upon the probabilities of metastasis of nervous disease, in the present case, and others, but merely make a statement of "unvarnished" facts, leaving your readers to draw their own conclusions therefrom; premising only thus far, that the case may be found to present points of interest, both in regard to the peculiarity of its commencement, and in presenting the diagnostic signs of a disease, in the illustration of which our standard medical literature, as far as I have referred, is somewhat deficient.

The first symptom which supervened upon the transfer of the disease inwardly, was *intense* and *persistent cephalalgia.*

This would generally come on in the morning and continue throughout the day and greater part of the night, and was the most constant symptom throughout, of the course of the disease. About the first of June, strabismus of a decided character occurred, the sense of hearing became slightly impaired, and partial hemiplegia followed, upon the opposite side. The latter symptom, however, was quite mild, being indicated merely by slight numbness and coldness over the right side. About this time vertigo and vomiting occurred occasionally, and the mind also became much enfeebled, thus heralding the impairment which was taking place in the cerebral functions. To use the expression of the parents, he became notional and silly in conversation.

Toward the close of the month, the patient, while riding horseback, over a mile from home, was seized with an epileptic attack, fell from his horse, and was taken up and carried home insensible. Soon after being placed in bed he partially revived; his mind, however, remained in a sort of haze for over half an hour afterward, his conversation being mostly incoherent, and his eye-sight only partly restored in the mean time.

I should have mentioned that when I first attended the patient, he resided in Wheeling, Cook county, Ill. About the first of June I moved to my present locality. Here I received a minute account of the progress of the case, and finally, in July, he was brought here for treatment. I was struck at once, on seeing him again, by his debilitated and cachectic appearance. Judging that supporting treatment was one of the indications to be fulfilled, I at once prescribed Syrup Ferri. Iodidi, with blisters behind the ear. The temporary effect of this treatment was even more favorable than I had ventured to expect it would be. All the symptoms gradually abated, until about the middle of September, when the patient appeared, with the exception of occasional vertigo, and a slight vestige of strabismus, entirely recovered. No relapse followed until about the beginning of February last, when, in consequence of getting wet the symptoms all recurred. Now, at times, he exhibited phenomena much resembling those of hysteria, appearing apprehensive, peevish, and difficult to please. His eyes became very sensitive to light; so much so that he would keep them closed for hours at a time, to avoid it. He was oppressed, much of the time, with that peculiar anxiety which is so often manifested upon the encroachment of disease upon the vital organs. In this condition he remained until the 22d, when a second epileptic attack closed the scene.

The best exposition which we have ever met with of cases of the above nature, is a monograph published by Freidreich,* containing the particulars of forty-five cases, eleven of which were observed by himself. I will present here an abstract from his article, showing briefly the symptoms, and the proportionate number of cases in which each presented itself, according to the locality of the tumor:

| SYMPTOMS. | LOCALITY OF TUMOR. |
|---|---|
| Obstinate cephalalgia. | Cerebrum, 18 cases—(14.)<br>Base of Brain, (9)—Frontal Headache, (13)<br>Petuitary 1, case 1.<br>Anterior base of brain, in all cases examined.<br>Peduncl. Cereb. and Cerebel.<br>Cerebellum, 8 cases—(7.) |
| Vertigo and Vomiting. | Cerebrum, (9.) |
| Derangement of motory functions, paralysis and epileptic convulsions. | Cerebrum, (14.)<br>Base of Craneum, (common.)<br>Peduncles, paralysis of face, opposite side. |
| Derangement of Special Senses. | Cerebrum, (10); Sight (7.)<br>Base of brain, sight, (7); hearing, (5); smell, (3.)<br>Pitutay case, 1 with double blindness.<br>Anterior part of base, 2 cases, symptoms same as above.<br>Pedim. des complex derang. spec. sense. |

* *Beiträge zur Lehne oon dem Geschwulsten innerhalb der Schädelhähle, Wurz. urg*, 1853.

| SYMPTOMS. | LOCALITY OF TUMOR. |
|---|---|
| Intelligence impaired. | Cerebrum, (11.)<br>Base of brain, (5.) |
| Occipital cephalalgia. | Cerebellum (4.)<br>This symptom, when it occurs, may be considered pathognomonic of tumor's location at this point. |

The numbers not inclosed in brackets denote the number of cases seen; those not inclosed, the number of times the symptom alluded to occurred. Strabismus is mentioned as common.

